# When the Cytoskeleton Fails: Primary Structural Defects Underlying Disorders with Neurological Involvement

**DOI:** 10.3390/ijms27198782

**Published:** 2026-09-30

**Authors:** Gerasimos Terzopoulos, Evangelos Axarloglou, Evangelie Sintou, Anna Makridou, Sofia Gargani, Soultana Meditskou, Maria Eleni Manthou, Paschalis Theotokis, Iasonas Dermitzakis

**Affiliations:** Department of Histology-Embryology, School of Medicine, Aristotle University of Thessaloniki, 54124 Thessaloniki, Greece; gterzop@auth.gr (G.T.); evanaxar@auth.gr (E.A.); evangelisintou@gmail.com (E.S.); annmak4@gmail.com (A.M.); sgargani@bio.auth.gr (S.G.); sefthym@auth.gr (S.M.); mmanthou@auth.gr (M.E.M.); ptheotokis@auth.gr (P.T.)

**Keywords:** cytoskeleton, neurogenetics, pathophysiology, microtubules, actin, intermediate filaments, neurodegeneration

## Abstract

Cytoskeletal disorders affecting the nervous system are a diverse group of diseases that display a wide variety of clinical manifestations. Mutations in genes encoding cytoskeletal components disrupt the structural and functional integrity of this complex network, impairing normal development and function of the central and peripheral nervous systems. There are also certain diseases which lead to progressive neurodegeneration, and others which result from an interplay of multiple molecular pathways. This review outlines the primary cytoskeletal disorders affecting the nervous system. To provide a comprehensive overview, these diseases are categorized by their predominantly affected cytoskeletal component—namely microtubules, actin filaments, intermediate filaments, and cytoskeleton-associated proteins. Each category highlights the broader pathophysiological context of the underlying defect rather than an isolated structural disturbance. A deeper understanding of the complex pathophysiology of these disorders may contribute to the discovery of potential therapeutic targets, since current medical management primarily provides symptomatic relief or slows their progression.

## 1. Introduction

The cytoskeleton of human cells is a complex, exquisitely orchestrated network of diverse proteins that mediates a wide spectrum of cellular functions, including cell division, cargo transport, signal transduction, cellular adhesion, motility, and shape modification [1]. The cytoskeleton is composed of three major filament systems: microtubules, actin filaments, and intermediate filaments [1,2]. Nonetheless, numerous other associated molecules drive dynamic shifts and mediate fine-tuned interplay among the different components of this cellular structure [1]. Microtubules are composed of α- and β- tubulin heterodimers and play a pivotal role in intracellular transport and cell shape modification [1,3]. They are also crucial components of the mitotic spindle during cell division [4]. Actin filaments are semi-flexible proteins that modulate the shape and participate in the adhesion and movement of cells [1,5]. On the other hand, intermediate filaments are a heterogeneous group of cytoskeletal components with multiple functions, such as organelle arrangement within cells, interaction with other cytoskeletal proteins, participation in apoptosis, adhesion and migration, as well as fortification of the cell against large stress [1,6,7]. Notably, there are four classes of cytoplasmic intermediate filaments, which include keratin, desmin, peripherin and glial fibrillary acidic protein among others, and one class of nuclear filaments, the lamins [1].

There is growing evidence highlighting the pathophysiologic interplay between cytoskeletal abnormalities and disorders with neurological features [8,9,10]. The three components of the neuronal cytoskeleton, namely microtubules, microfilaments (actin) and neurofilaments (intermediate filaments), compose a meshwork which contributes to the development of axons during embryogenesis, the proper function of neurons, as well as the induction of neuroplasticity and nerve regeneration [11,12,13,14]. Interestingly, strong evidence suggests that neurodegeneration is precipitated by mutations in genes encoding cytoskeletal proteins [9]. For instance, disruption of the microtubules can directly impact the neuronal development, since they actively participate in cell division, migration and axonal transport [15,16]. Microfilament disorganization leads to defective cellular structure and loss of integrity of the affected tissues [9]. Regarding neurofilaments, potential defects can hinder the cellular architecture and significantly reduce cells’ durability under mechanical stress [17]. Worth mentioning examples of disorders in which cytoskeletal abnormalities are the primary cause include lissencephaly 3, hypomyelinating leukodystrophy 6, Baraitser-Winter syndrome, Alexander disease, and giant axonal neuropathy [18,19,20,21,22]. Each of these disorders is caused by distinct mutations in specific cytoskeletal proteins, resulting in different phenotypes and characteristic manifestations.

In the era of multi-omics data and precision medicine, there is a growing shift toward a more pathophysiology-oriented organization of clinical entities [23,24,25]. Similar mechanistic approaches have recently been applied to neurological disorders associated with mitochondrial, lysosomal, and neural crest dysfunction, emphasizing the value of organizing heterogeneous diseases according to their primary cellular context rather than phenotype alone [26,27,28]. Although previous reviews have addressed individual cytoskeletal compartments, protein families, specific disease groups, or the cytoskeleton broadly in human disease—including actinopathies, intermediate-filament disorders, tubulinopathies, neurofilament-related diseases, neurodegeneration, and neurodevelopment—an integrated framework encompassing the major cytoskeletal systems involved in neurological disease remains lacking [8,29,30,31,32,33,34,35,36,37,38]. Thus, the present review aims to present the primary disorders of the cytoskeleton affecting the nervous system. From a clinical perspective, this mechanistic understanding may improve disease recognition, support genotype-phenotype correlations, and highlight potential therapeutic targets, particularly in disorders for which current management remains largely symptomatic.

## 2. Review Methodology

To construct a focused, coherent review, we adopted a structured, disease-centered selection strategy to identify representative disorders in which cytoskeletal dysfunction is a primary driver of pathogenesis and is associated with neurological features. PubMed and Scopus were searched for studies describing genetic diseases linked to pathogenic variants in genes encoding cytoskeletal proteins or proteins directly involved in cytoskeletal organization, stability, dynamics, or intracellular transport. The objective was not to compile an exhaustive list of all conditions in which cytoskeletal abnormalities have been reported, but to select representative disease entities that best illustrate the major mechanistic categories through which primary cytoskeletal disruption affects the nervous system. Selection was therefore guided by four main criteria: the presence of neurological features; evidence that the cytoskeletal defect represents a primary pathogenic mechanism rather than a secondary downstream consequence; availability of mechanistic or genotype–phenotype data linking the affected protein to cytoskeletal function; and recognition of the condition as a defined disease entity in the OMIM catalogue.

**Figure 1:**
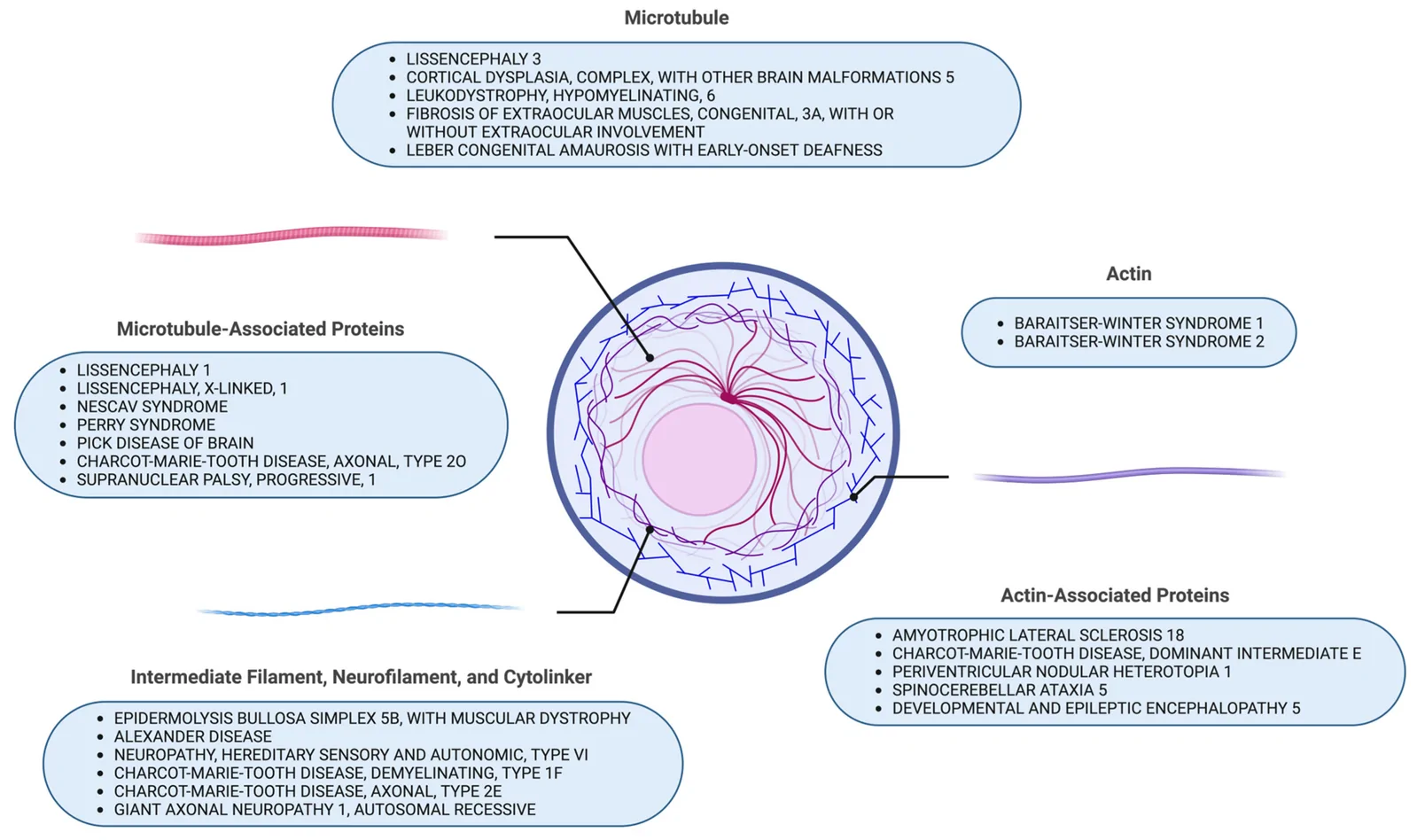
Schematic overview of the major primary cytoskeletal disorders with neurological features. Diseases are categorized according to the cytoskeletal compartment primarily affected by the underlying disease mechanism, including microtubules, microtubule-associated proteins, actin, actin-associated proteins, intermediate filaments, neurofilaments, and cytolinker proteins. Pink, purple, and blue lines represent microtubules, actin filaments, and intermediate/neurofilaments, respectively. Created in BioRender. Dermitzakis, I. (2026). https://BioRender.com/ey51xt4.

Special attention was given to disease-level specificity, with included conditions cross-checked against OMIM to ensure that the review addressed distinct disease entities rather than broad clinical spectra, nonspecific syndromic descriptions, or isolated imaging findings. Disorders were considered eligible when neurological involvement formed part of the recognized phenotype, even if they were not exclusively neurological, whereas conditions in which cytoskeletal abnormalities were secondary or incidental were excluded. The selected diseases were subsequently organized according to the predominantly affected cytoskeletal system, distinguishing primary microtubule defects from microtubule-associated protein disorders, primary actin defects from actin-associated protein disorders, and conditions involving intermediate filaments, neurofilaments, or cytolinker proteins. Given the substantial clinical, phenotypic, and mechanistic heterogeneity of cytoskeleton-related neurological disorders, including diversity within individual cytoskeletal compartments, this organization should not be interpreted as a formal nosological or diagnostic classification or as evidence of a shared pathogenic pathway. Rather, it provides a pathophysiology-oriented framework for presenting representative OMIM-defined disorders within their most relevant cytoskeletal context and examining how primary cytoskeletal defects contribute to neurological disease. This organizational scheme is summarized in Figure 1.

## 3. Microtubule System and Associated Proteins in Disorders with Neurological Features

### 3.1. Primary Microtubule Defects

Structural tubulinopathies are disorders caused by mutations in genes encoding α- and β-tubulin subunits that lead to abnormalities in microtubule assembly, dynamics, and interactions with molecular motors. This results in defects in neuronal migration, cortical development, axon guidance, myelination, intracellular transport, and ciliary function, which lead to a wide spectrum of neurological and sensory phenotypes [39]. Disorders discussed in this category are lissencephaly 3 (LIS3), cortical dysplasia, complex, with other brain malformations 5 (CDCBM5), leukodystrophy, hypomyelinating, 6 (HLD6), fibrosis of extraocular muscles, congenital, 3a, with or without extraocular involvement (CFEOM3A), and Leber congenital amaurosis with early-onset deafness (LCAEOD). All the above diseases are listed in Table 1.

LIS3 (OMIM: 611603) is caused by a mutation in the alpha-tubulin 1A (*TUBA1A*) gene on chromosome 12q13 [18,45]. These mutations in *TUBA1A* can impair α-tubulin folding and heterodimer assembly, alter microtubule dynamics, and disrupt interactions with microtubule-associated proteins and motor proteins such as dynein. These changes interfere with neuronal migration, resulting in defective cortical lamination and abnormal brain development [52,53]. Neuropathological findings indicate that TUBA1A-related disease is not limited to migratory failure. Reduced numbers of MAP2-positive cortical neurons, together with callosal agenesis and corticospinal tract disorganization, lead to impaired neuronal differentiation and axonogenesis [55]. Affected individuals almost universally exhibit severe-to-profound intellectual disability with significant motor impairment—including severe developmental delay or spastic tetraplegia—frequent early-onset, drug-resistant epilepsy, and congenital or postnatal microcephaly [54,55]. Most cases of LIS3 result from *de novo* mutations and follow an autosomal dominant inheritance pattern [18,53].

CDCBM5 (OMIM: 615763) is an autosomal dominant disease caused by mutations in the gene that codes the tubulin beta-2A chain (*TUBB2A*) [44]. The gene is located on chromosome 6p25 [39,43,45]. *De novo* mutations in the *TUBB2A* gene hinder its interaction with alpha-tubulin-bound GTP, therefore disrupting the formation of stable alpha/beta-tubulin heterodimers. This functional failure disrupts microtubule polymerization and the essential mechanical tasks it performs during brain development, resulting in abnormalities such as pachygyria and simplified gyral patterning [42,43]. Phenotypic features of CDCBM5 typically include severe global developmental delay, seizures, hypotonia, and a thin *corpus callosum*, with onset in early infancy [42,43,44].

HLD6 (OMIM: 612438), formerly known as hypomyelination with atrophy of the basal ganglia and cerebellum, is associated with the *TUBB4A* gene located on chromosome 19p13 [19,45]. In HLD6, *TUBB4A* mutations disrupt β-tubulin dimer formation and microtubule dynamics, impairing oligodendrocyte survival, myelin formation, and neuronal transport, particularly in the cerebellum and basal ganglia. These combined defects produce the characteristic hypomyelinating leukodystrophy phenotype [46,47,48]. More specifically, a commonly found mutation, p.Asp249Asn, replaces aspartic acid with asparagine at position 249, results in improper microtubule assembly that leads to impairment in both neurons and oligodendrocytes, including neurons in the putamen and cerebellum. Because the transport of proteolipid protein 1 (PLP1) and myelin basic protein (MBP) messenger RNA to the myelin sheath depends on microtubules, TUBB4A dysfunction may lead to fewer branches of oligodendrocytes and insufficient transportation of the necessary components of myelin, causing hypomyelination [47]. The neurological phenotype includes progressive spasticity, dystonia, and hypomyelination visible on MRI. Inheritance of HLD6 is autosomal dominant, most commonly arising *de novo* and typically presenting as a sporadic disorder [39,45].

CFEOM3A (OMIM: 600638) is a congenital cranial dysinnervation disorder associated with the *TUBB3* gene on chromosome 16q24. Although it is most commonly mapped to the FEOM3 locus on chromosome 16, it can also be rarely caused by mutations in the KIF21A gene, the primary gene associated with CFEOM1 [40]. Its mechanism involves perturbation of microtubule dynamics and kinesin-1 motor interactions, which are critical for axon guidance and maintenance of ocular nerves [41]. The phenotype consists of highly variable unilateral or bilateral ophthalmoplegia and blepharoptosis resulting from oculomotor nerve dysinnervation that notably spares pupillary function, as well as synkinetic jaw movements. It can occur with or without cortical dysplasia [40]. Inheritance is autosomal dominant [39,40].

LCAEOD (OMIM: 617879) is a very rare autosomal dominant disorder due to a heterozygous mutation in the *TUBB4B* gene [49,51]. The mutated gene encodes a variant of beta-tubulin, a protein found at high concentrations in the retina, brain, testis, and bone marrow. This alteration leads to impaired motility and sensory function of motile and sensory cilia. The phenotype depends on the specific beta-tubulin variant that is produced and includes early-onset sensorineural hearing loss and endocochlear deafness, accompanied by retinal degeneration and progressive cone dystrophy [50]. Other documented features are nystagmus, hyperopia, reduced visual acuity and photophobia [51].

### 3.2. Impairment of Microtubule-Associated Proteins and Neuronal Transport

**Figure 2:**
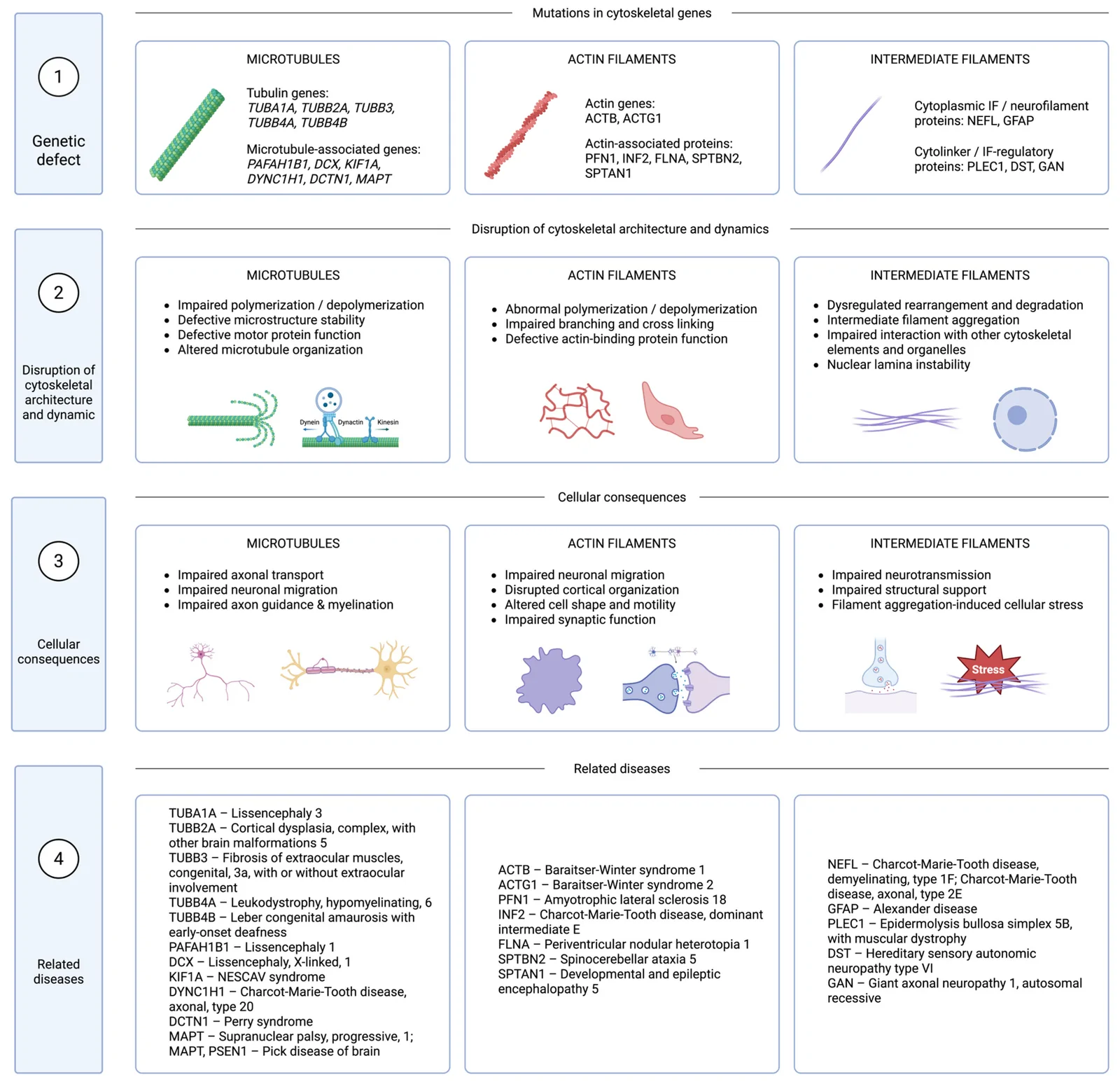
Pathophysiological framework linking cytoskeletal gene defects to neurological disease. The schematic illustrates the sequential relationship between pathogenic variants in genes encoding microtubule, actin, and intermediate filament components or associated proteins, disruption of cytoskeletal architecture and dynamics, the resulting cellular consequences, and representative related disorders. Diseases are grouped according to the cytoskeletal compartment predominantly affected by the underlying disease mechanism. Created in BioRender. Dermitzakis, I. (2026). https://BioRender.com/485kv06.

Neuronal migration and MAP-related disorders stem from defects in microtubule-associated proteins or proteins that regulate microtubule stability and nucleokinesis [59]. Disorders discussed in this category include lissencephaly 1 (LIS1), lissencephaly, X-linked, 1 (LISX1), Charcot-Marie-Tooth disease, axonal, type 2O (CMT2O), Perry syndrome, NESCAV syndrome, supranuclear palsy, progressive, 1 (PSNP1), and Pick disease of the brain (Figure 2).

The identified gene/protein most commonly involved in LIS1 (OMIM: 607432) is *PAFAH1B1* on chromosome 17p13. This protein interacts with dynein and regulates nucleokinesis during radial and non-radial migration; haploinsufficiency causes impaired or slowed neural migration [56]. The delay may prevent these neurons from reaching their targets during critical periods of brain-circuit formation, helping to explain the association between abnormal interneuron positioning and epilepsy [56]. LIS1 phenotype is characterized by an agyric or pachygyric brain surface, microcephaly, cerebellar hypoplasia, and dysmorphic basal ganglia. It is a spectrum extending from complete agyria to subcortical band heterotopia (SBH) [57]. SBH lies at the milder end of the lissencephaly malformation spectrum [58]. In contrast, the more pronounced phenotypes, including isolated type 1 lissencephaly and, at the most severe end of the spectrum, Miller–Dieker syndrome, are characterized by severe pachygyria/agyria with a posterior-to-anterior gradient, intellectual disability, and epilepsy [59]. Inheritance is primarily autosomal dominant [57].

LISX1 (OMIM: 300067) is caused by a mutation in the doublecortin gene (*DCX*) on the X chromosome. Doublecortin stabilizes microtubules and can link them to actin filaments [60]. A mutation in the *DCX* gene results in the impairment of cortical neurons to perform radial migration [60,61]. The condition is phenotypically expressed with a band of heterotopic grey matter situated under the white matter, with clinical severity depending on the band’s thickness [62]. The most common clinical symptoms include classic lissencephaly in males and a less pronounced clinical presentation in females termed subcortical band heterotopia (also known as subcortical laminar heterotopia or double cortex syndrome) [62]. Inheritance is X-linked; thus, classic LISX1 is a predominantly male phenotype, whereas females more often have the SBH phenotype [62].

Charcot-Marie-Tooth (CMT) neuropathies are a diverse group of inherited peripheral nervous system diseases [84]. They are divided into two major categories based on electrophysiological findings: CMT1 and CMT2 [85,86]. CMT1 mainly includes demyelinating neuropathies, which display reduced nerve conduction velocity (NCV), while CMT2 includes axonal neuropathies, which are associated with reduced compound muscle action potential and normal or slightly decreased NCV [85,86]. Nonetheless, there are certain types of CMT disease with features of both demyelination and axonopathy (intermediate CMT). The onset of symptoms ranges from childhood to adulthood. Severity of symptoms, namely muscle atrophy and weakness due to peripheral nerve degeneration, may also vary [85,86].

Charcot-Marie-Tooth disease, axonal, type 2O (CMT2O) (OMIM: 614228) belongs to the inherited axonal neuropathies of the Charcot-Marie-Tooth disease spectrum and follows the autosomal dominant pattern of inheritance. The mutated gene, *DYNC1H1*, normally encodes the cytoplasmic dynein heavy chain 1 [67]. As is well known, dynein is a cytoskeletal motor protein closely associated with microtubules, as it mediates the retrograde transport of cargo within cells towards the minus end of microtubules [66]. *DYNC1H1* mutations directly disrupt cytoskeletal integrity, as evidenced by histopathological findings consistent with axonal degeneration [67]. CMT2O patients typically present with delayed motor milestones, distal lower limb weakness, paresthesias and neuropathic pain. However, the upper limbs are affected less commonly than in other subtypes of the disease, and ambulation is preserved into adulthood [67].

Perry syndrome (OMIM: 168605) refers to an autosomal dominant neurodegenerative disorder caused by pathogenic mutations in *DCTN1*, a gene coding for dynactin, on chromosome 2p12-14. These mutations reduce microtubule binding, thus hindering the initiation of retrograde transport at distal neurite tips. Perry syndrome variants occur in a microtubule-binding region of p150Glued, a protein within the dynactin complex. These variants weaken dynactin’s attachment to microtubules and related proteins, reducing its accumulation at the ends of axons and its ability to recruit the motor protein dynein [68]. Furthermore, the impaired dynactin function causes the TDP-43 protein (transactive-response DNA-binding protein of 43 kDa) to aggregate into toxic clumps called neuronal intracytoplasmatic inclusions, which precipitate nuerodegeneration [68,69]. As described in 1975 [70], the syndrome consists of parkinsonism and depression in adults, followed by weight loss and hypoventilation. There have been expansions in the classic presentation to include features resembling other neurodegenerative disorders, particularly frontotemporal dementia and progressive supranuclear palsy [71,72]. Considerable phenotypic differences could even occur within affected family members [71].

Another microtubule-associated neurodegenerative disorder is NESCAV syndrome (OMIM: 614255). The gene involved in the development of the syndrome is *KIF1A*, and the inheritance pattern is autosomal dominant [63]. *KIF1A* normally transports synaptic-vesicle precursors and other cargos along axons, including vesicles containing alpha-amino-3-hydroxy-5-methyl-4-isoxazolepropionic acid (AMPA) receptors, which support synaptic transmission and plasticity [65]. NESCAV syndrome is caused by *de novo* heterozygous missense mutations in the *KIF1A* motor domain that impair ATP-dependent axonal transport, reduce distal neurite localization, and likely exert a dominant-negative effect on *KIF1A* dimers [64,65]. The neurological phenotype is variable and comprises a diverse range of clinical symptoms such as global developmental delay, walking impairment due to progressive spasticity and intellectual disability [63].

Tau protein is another major microtubule-associated protein. It is encoded by the microtubule-associated protein tau (*MAPT*) gene on chromosome 17q21 and forms a scaffold for microtubule polymerization and stabilization, and participates in signal transduction, DNA/RNA protection, and synaptic function [73,74]. Therefore, mutations causing disarrangement of the tau protein can eventually lead to a pathological phenotype. PSNP1 is an autosomal dominant, primary tauopathy and a common cause of degenerative Parkinsonism [80,83]. The pathophysiology includes hyperphosphorylated 4R-tau isoforms that accumulate in subcortical neurons, thereby creating neurofibrillary tangles, and in glial “tufted astrocytes”, which disrupt microtubule-mediated neuronal architecture [74,75,81]. These are active causes of neurodegeneration. Common symptoms of PSNP1 are Parkinsonism, supranuclear gaze palsy, postural instability, axial rigidity, and dysarthria [74,75,82]. Although PSNP1 is predominantly sporadic, the H1 haplotype is strongly associated with increased disease susceptibility, supporting the contribution of tau protein and microtubule dysfunction to its pathogenesis [74,75,80].

Pick disease of brain (OMIM: 172700) is a primary structural tauopathy associated with mutations in the *MAPT* gene (e.g., K257T) and sporadic risk factors. These pathogenic mechanisms promote the aggregation of 3R-tau into round, argyrophilic “Pick bodies” within neurons and “Pick Cells”, which are neurons with increased size [73,74,75]. This causes degeneration of the neuronal cytoskeleton, thus resulting in severe shrinkage of the frontal and temporal lobes, behavioral disinhibition, social comportment disorder, and progressive non-fluent aphasia [74,76,78]. Although sporadic, Pick disease of brain has an autosomal-dominant inheritance pattern in family cases [73,74,75].

## 4. Actin Cytoskeleton and Actin-Associated Proteins in Disorders with Neurological Involvement

### 4.1. Primary Actin Impairment

Actin proteins constitute the fundamental structural elements of the actin cytoskeleton and participate in numerous cellular processes, including cell migration, maintenance of cell shape, and tissue morphogenesis [87]. During neurodevelopment, actin dynamics are critical for neuronal migration, growth cone behavior, and cortical organization [88]. This is accomplished via the interplay between actin and myosin filaments [87]. In nonmuscle cells, this network is much less ordered yet more dynamic compared to striated muscle [87]. The interaction of actin and myosin filaments produces contractile forces in cells, eventually contributing to cell division, migration, cell-shape modifications [87]. More specifically, during neuronal development, an actin-based contractile ring mediates the separation of the newly formed neuron from its progenitor cell, which is followed by actin-driven cell migration throughout the brain [89]. Pathogenic variants affecting the actin genes themselves represent a distinct subgroup of cytoskeletal disorders, often characterized by complex developmental phenotypes involving the nervous system and other organ systems [87]. Disorders discussed in this category include Baraitser-Winter syndrome 1 (BRWS1) and 2 (BRWS2).

BRWS1 (OMIM: 243310) is caused by heterozygous pathogenic variants in *ACTB*, which encodes β-actin, and is inherited in an autosomal dominant manner [90,91]. Most cases arise *de novo*. β-actin is a major component of the actin cytoskeleton and is essential for cell motility, neuronal migration, and morphogenesis during brain development [20]. Disease-causing variants alter filament dynamics, hindering actin polymerization and stability [90]. The recurrent ACTB (actin-beta) p.Arg196His variant is associated with a reduction in filamentous actin and cellular stiffness. It also weakens the attachment of cortical actin to the plasma membrane [20]. Furthermore, the variant causes disruption in actin reorganization mediated by the actin-related protein 2/3 (Arp2/3) complex and cofilin. This may impair dendritic-spine maturation, while reduced cell proliferation and migration may contribute to cortical malformations [20]. Clinically, affected individuals present with characteristic craniofacial features, ptosis and/or coloboma, developmental delay or intellectual disability, epilepsy, and structural brain abnormalities [20,90,91].

Similarly, BRWS2 (OMIM: 614583) is caused by heterozygous pathogenic variants in *ACTG1*, which encodes γ-actin and is inherited in an autosomal dominant manner [90,91]. Like *ACTB*, *ACTG1* participates in regulate actin cytoskeletal architecture and contributes to neuronal migration and tissue morphogenesis [90]. Disease-causing variants impede cells’ ability to repair defects in the actin filaments, thus undermining the cellular structural integrity s [90,92]. The phenotype overlaps substantially with BRWS1 and includes craniofacial dysmorphism, ocular anomalies, developmental delay, epilepsy, and cortical malformations [90]. These clinical manifestations overlap with many other neurodevelopmental disorders; placement in the actin category reflects the causal ACTB/ACTG1 defect rather than a phenotype specific to actin dysfunction.

### 4.2. Actin-Associated Protein Defects

The function of the actin cytoskeleton depends on a diverse group of associated proteins that regulate actin polymerization, crosslink actin filaments, anchor them to membrane-associated complexes, or integrate them with other cytoskeletal networks [93]. Disruption of these proteins affects not only the structural integrity of cells but also intracellular signaling, neuronal migration, axonal maintenance, and synaptic function [93]. Consequently, pathogenic variants in actin-associated proteins give rise to a broad spectrum of neurological disorders encompassing neurodevelopmental diseases, neurodegeneration, epilepsy, and peripheral neuropathies. This group also includes proteins involved in the spectrin–actin membrane skeleton, a specialized neuronal structure critical for membrane stability and axonal organization [94]. Disorders discussed in this category include amyotrophic lateral sclerosis 18 (ALS18), Charcot-Marie-Tooth disease, dominant intermediate E (CMTDIE), periventricular nodular heterotopia 1 (PVNH1), spinocerebellar ataxia 5 (SCA5), and developmental and epileptic encephalopathy 5 (DEE5).

Amyotrophic lateral sclerosis (OMIM: 614808) is considered a genetically heterogeneous disease, comprising distinct molecular subtypes that arise from pathogenic variants in different genes [95]. ALS18 is caused by heterozygous mutations in *PFN1*, encoding profilin-1. The disorder follows an autosomal dominant pattern of inheritance [96,97]. Profilin-1 is an actin-binding protein that regulates actin monomer availability and filament assembly; mutant proteins disturb actin dynamics and broader cytoskeletal homeostasis in motor neurons. The phenotype is familial adult-onset amyotrophic lateral sclerosis with progressive upper and lower motor neuron degeneration, weakness, and respiratory decline [96].

In addition to neurodegenerative phenotypes, defects in actin-associated proteins may also result in peripheral neuropathies. CMTDIE (OMIM: 614455) is an autosomal dominant subtype of CMT disease with overlapping elements of both axonal and myelin degradation. It is caused by mutations in the *INF2* gene [98]. The encoded protein is an endoplasmic-reticulum-associated formin protein abundantly expressed in Schwann cells. Its primary function is the remodeling of actin and microtubule cytoskeletons. Evidence shows that mutations of the *INF2* gene lead to a reduction in long actin stress fibers and microtubule dysregulation. Moreover, abnormal function of the INF2 (inverted formin 2) protein impairs protein targeting to the plasma membrane, thus disrupting proper myelination of neurons [98]. Under the microscope, there is a combination of axonal and demyelinating changes, namely a decrease in myelinated fibers and the formation of onion bulbs, which consist of flattened Schwann cell cytoplasm processes [98,99]. The onset of neurological symptoms varies from childhood to adulthood, and severity ranges from moderate to severe peripheral nervous system impairment. The patients present with gait disturbance, muscle atrophy, diminished deep tendon reflexes and moderate sensorineural hearing loss [98]. Interestingly, the INF2 protein is also expressed in the podocytes of the kidney glomerulus, and *INF2* mutations directly affect renal function. Patients usually develop proteinuria and progressive renal failure. Biopsy findings are consistent with focal segmental glomerulosclerosis [98].

The importance of actin-associated proteins in neuronal migration is further illustrated by PVNH1 (OMIM: 300049), which is caused by mutations in *FLNA*, encoding filamin A, and is inherited in an X-linked dominant manner [100]. Filamin A is an actin-binding and actin-crosslinking protein that supports cytoskeletal integrity, neuronal migration, dendritic maturation, and synaptic development [101]. Disruption of *FLNA* impairs neuronal migration, producing nodules of ectopic grey matter along the lateral ventricles [101]. The phenotype commonly includes epilepsy, periventricular nodular heterotopia, variable cognitive involvement, and sometimes vascular or connective-tissue manifestations [100].

Beyond classical actin-binding proteins, disturbances of the spectrin–actin membrane skeleton represent another important pathogenic mechanism. SCA5 (OMIM: 600224) is an autosomal dominant disease caused by heterozygous variants in *SPTBN2*, encoding β-III-spectrin [102]. β-III-spectrin is part of the neuronal spectrin–actin membrane skeleton and is highly relevant to Purkinje-cell function; the underlying molecular defect can alter actin binding, membrane-skeleton organization, and trafficking of neuronal membrane proteins [102]. The phenotype is usually slowly progressive cerebellar ataxia with gait ataxia, limb incoordination, dysarthria, and ocular motor abnormalities [103].

Whereas *SPTBN2*-associated disease primarily affects cerebellar function, pathogenic variants in other spectrin family members lead to severe developmental epileptic disorders [104]. DEE5 (OMIM: 613477) is caused by heterozygous alterations in *SPTAN1*, encoding αII-spectrin [105]. αII-spectrin is a major neuronal membrane-skeleton protein that supports axonal organization, membrane stability, and neuronal excitability [105]. It is inherited in an autosomal dominant manner, and most reported cases arise *de novo* [105]. Mutant proteins disrupt spectrin network integrity and neurodevelopmental organization [104]. The clinical spectrum consists of early-onset epileptic encephalopathy, infantile spasms or refractory seizures, severe developmental delay, microcephaly, hypomyelination, cerebral/cerebellar or brainstem atrophy, and spasticity [104,105]. All the above diseases are presented in Table 2.

## 5. Intermediate Filament, Neurofilament, and Cytolinker Disruption

This category involves proteins that bind intermediate filaments to other cellular structures. Mutations altering the normal structure and function of these cytoskeletal components jeopardize the cellular integrity and impair vital neuronal processes, namely axonal transport and neurotransmission [29,30,31]. Subsequently, the genetic alterations lead to various central and peripheral nervous system symptoms, as well as extra-neuronal manifestations which are characteristic of each disorder. This section includes epidermolysis bullosa simplex 5B, with muscular dystrophy (EBS5B), Alexander disease, neuropathy, hereditary sensory and autonomic, type VI (HSAN VI), Charcot-Marie-Tooth disease, demyelinating, type 1F (CMT1F), Charcot-Marie-Tooth disease, axonal, type 2E (CMT2E) and Giant axonal neuropathy 1, autosomal recessive (GAN1).

EBS5B (OMIM: 226670) is caused by the abnormal gene known as *PLEC1* that encodes the protein plectin [106,107]. Plectin is a high-molecular-weight linker protein that binds intermediate filaments, such as keratin in skin and desmin in muscle, to the plasma membrane and hemidesmosomes [108]. Thus, the broad-range relevance of cytoskeletal integrity is evident in the skin as well, where keratin networks, epidermal differentiation programs, stem-cell dynamics, and molecular homeostasis collectively preserve tissue architecture and mechanical resilience [109,110,111]. The corresponding mutations cause collapse of the intermediate filament network, resulting in structural fragility and mitochondrial abnormalities [112]. The hallmark of the disease is the formation of hemorrhagic blisters, along with skin and oral mucosa erosions. Neurologic symptoms include congenital myasthenic syndrome, characterized by muscle weakness because of malfunctioning neuromuscular junctions. Other documented features include brain atrophy, delayed motor skills (walking at age four years), and psychiatric issues such as aggression or depression [108]. It is inherited in an autosomal recessive fashion [31,108].

A phenomenon that is also worth examining is structural filament impairment in astrocytes. Astrocytes are abundant glial cells and act as the central nervous system’s support system [31,113,114]. One of the most notable examples of structural filament dysfunction in astrocytes is Alexander disease (OMIM: 203450), in which the affected gene/protein is glial fibrillary acidic protein (GFAP). Mutations lead to the aberrant accumulation of intermediate filament-rich aggregates known as Rosenthal fibers within the central nervous system [21]. This accumulation is a source of cellular stress and potentially cell death. The neurological phenotype includes early-onset megalencephaly, progressive spasticity, and dementia [115]. It is inherited in an autosomal dominant manner [116].

HSAN VI (OMIM: 614653) is an autosomal recessive disease caused by *DST* mutations that impair dystonin, a protein critical for axonal cytoskeleton maintenance [117]. This protein stabilizes cellular structures by anchoring intermediate filaments (e.g., keratin in epithelial cells, neurofilaments in neurons) to other cellular components, such as desmosomes and hemidesmosomes [117]. Mutations within the neuronal isoforms result in disorganization of the actin cytoskeleton, leading to impairment of cell adhesion, spreading, and migration. For instance, mutations such as p.Ala203Glu prevent the protein from binding to actin [117]. The disease is clinically manifested by progressive distal sensory loss, severe pain insensitivity, painless fractures, and autonomic dysfunction such as decreased perspiration and pupillary abnormalities [118].

CMT1, as already mentioned, is considered a demyelinating neuropathy rather than a primary cytoskeletal disorder. Nonetheless, a specific subtype, CMT1F (OMIM: 607734), is primarily linked to mutations in the neurofilament light chain (*NEFL*) gene and follows the autosomal dominant or recessive pattern of inheritance, depending on the type of mutation [119]. Neurofilaments are essential for maintaining the structural integrity of neurons, particularly in large, myelinated axons. Neurofilament proteins are composed of three subunits: neurofilament light chain, neurofilament medium chain, and neurofilament heavy chain [30]. Mutations in the *NEFL* gene disrupt the assembly and stability of neurofilaments, which impair axonal transport and neurotransmission. This phenomenon impairs the delivery of vital proteins and organelles to nerve terminals [119]. Additionally, there is notable dysregulation microtubule dynamics and reduction in structural axonal support [119]. In the case of CMT1F, the primary defect in the cytoskeleton leads to subsequent demyelination [119]. CMT1F has an onset in infancy or childhood, with multiple PNS symptoms, namely progressive distal muscle weakness and wasting, sensory loss, pes cavus, and diminished deep tendon reflexes [119,120].

In contrast to CMT1F, CMT2E (OMIM: 607684), which is also caused by mutations in the *NEFL* gene, is only characterized by axonal damage. It is inherited in an autosomal dominant pattern and causes primary axonopathy [121,122]. Microscopic observations reveal the presence of giant axons that contain disorganized aggregates of neurofilaments. Other secondary findings include sporadic atrophic axons, while the density of myelinated fibers is preserved [122]. Classic symptoms of gait disturbance, muscle wasting, deformities of the feet (pes cavus) compose the phenotype of the disease [121].

GAN1 (OMIM: 256850) is an autosomal recessive neurodegenerative disease caused by mutations in *GAN* gene, which encodes the protein gigaxonin [22,123]. Under normal conditions, gigaxonin controls the rearrangement of intermediate filaments and contributes to the formation of a ubiquitin-ligase complex, which in turn regulates the degradation of intermediate filaments. Neuropathological examination of the nerves of patients has revealed aggregation of intermediate filaments [22]. These tangles of fibers interfere with neuronal mechanical properties, hinder signal transduction, and disrupt autophagy [124]. Symptoms begin in early childhood. First signs include both proximal and distal muscle weakness along with ataxia and distal sensory impairment, leading to gait instability. The patients are wheelchair-bound within 4 to 6 years of symptom onset [22,125]. During the course of the disease, individuals may present with dysarthria, dysphagia, and eventually develop respiratory failure in the second or third decade of life. CNS is also affected, leading to cerebellar dysfunction, optic atrophy and intellectual disability [22,125]. Furthermore, the patients have characteristic features, including high foreheads, curled hair, ichthyosis and keratosis pilaris. All the above diseases are listed in Table 3.

## 6. Conclusions

Cytoskeletal disorders with neurological involvement represent a heterogeneous group of diseases in which disruption of structural cellular components profoundly affects nervous system development and function. The present review organizes representative disorders by the primarily affected cytoskeletal elements—microtubules, microtubule-associated proteins, actin, actin-associated proteins, intermediate filaments, neurofilaments, and cytolinker proteins—to provide a pathophysiology-oriented framework and to highlight that these conditions should not be approached only as isolated clinical entities, but also within the cytoskeletal context relevant to their pathogenesis. Across these categories, mutations disturb essential processes such as neuronal migration, cortical organization, axonal transport, myelination, synaptic function, cellular stability, and neurodegenerative pathways. Nevertheless, establishing a direct association between gene mutations and clinical manifestations is difficult and may lead to precarious conclusions. This is because numerous molecular processes intervene between genotype and phenotype, leading to significant phenotypic variance and overlap among the clinical presentations of the disorders. Despite this complexity, the shared mechanistic denominator remains the primary cytoskeletal defect; thus, by focusing on primary cytoskeletal dysfunction rather than secondary downstream abnormalities, this review underscores the central role of cytoskeletal integrity in maintaining neurological structure and function.

This pathophysiology-based perspective may also support future mechanistic and therapeutic studies. A clearer understanding of how each cytoskeletal system contributes to disorders with neurological involvement could help identify disease-specific molecular vulnerabilities and refine the interpretation of genotype–phenotype relationships. The distinction between primary defects of cytoskeletal proteins and defects of cytoskeleton-associated regulators is particularly important, because it allows disease mechanisms to be analyzed within their most relevant structural and functional context. Although current medical management mainly provides symptomatic relief or slows disease progression, a deeper mechanistic understanding may contribute to the discovery of potential molecular therapeutic targets. In the era of precision medicine, gene therapy is undoubtably considered the ideal approach for each condition, since it directly targets the specific mutated genetic locus. However, gene therapy manufacturing is costly, complex and with limited accessibility. Furthermore, it requires appropriate vectors capable of crossing the blood–brain barrier and efficiently delivering the normal gene to the CNS [126]. 

Thus, instead of targeting the primary cause, there should also be attempts to facilitate the normal function of the specific dysregulated cellular mechanism. For instance, certain molecules that modulate microtubule dynamics have been considered as pharmacological therapies in neurodegenerative disorders [127]. Agents broadly used in oncology, such as taxols and epothilone B, act as microtubule stabilizers [127]. In vivo studies over the years have yielded promising results in terms of ameliorated behavioural and cognitive function [127,128,129,130]. Other potential therapeutic agents, namely davutenide, an oligopeptide, and noscapine, preserve and boost axonal transport in the context of neurodegenerative diseases [127,131,132]. Future work should therefore continue to examine these disorders through cytoskeleton-based categories, integrating clinical, genetic, histological, cellular, and molecular data where available. Such an approach may improve the systematic analysis of rare neurological diseases, facilitate comparison of disease-specific mechanisms across clinically heterogeneous phenotypes, and provide a stronger foundation for developing more targeted strategies in disorders driven by primary cytoskeletal disruption.

## Figures and Tables

**Table 1:** Primary cytoskeletal disorders involving microtubules and microtubule-associated proteins with neurological manifestations.

Disease	OMIM	Gene(s)	Inheritance	Clinical Features	References
Fibrosis of extraocular muscles, congenital, 3a, with or without extraocular involvement	600638	*TUBB3*	AD	Unilateral or bilateral ophthalmoplegia; Blepharoptosis; Synkinetic jaw movements	[39,40,41]
Cortical dysplasia, complex, with other brain malformations 5	615763	*TUBB2A*	AD	Global developmental delay; Seizures; Hypotonia	[39,42,43,44,45]
Leukodystrophy, hypomyelinating, 6	612438	*TUBB4A*	AD	Progressive spasticity; Dystonia; Microcephaly; Gait impairment; Seizures; Tremor; Poor speech development	[19,39,45,46,47,48]
Leber congenital amaurosis with early-onset deafness	617879	*TUBB4B*	AD	Early-onset sensorineural hearing loss; Endocochlear deafness; Retinal degeneration; Progressive cone dystrophy; Nystagmus; Hyperopia; Reduced visual acuity; Photophobia	[49,50,51]
Lissencephaly 3	611603	*TUBA1A*	AD	Intellectual disability; Motor impairment; Drug-resistant epilepsy; Congenital or postnatal microcephaly	[18,45,52,53,54,55]
Lissencephaly 1	607432	*PAFAH1B1*	AD	Lissencephaly; Microcephaly; Severe pachygyria/agyria; Intellectual disability; Epilepsy; Subcortical band heterotopia	[56,57,58,59]
Lissencephaly, X-linked, 1	300067	*DCX*	XL	Classic lissencephaly in males; Subcortical band heterotopia in females	[60,61,62]
NESCAV syndrome	614255	*KIF1A*	AD	Global developmental delay; Walking impairment; Intellectual disability	[63,64,65]
Charcot-Marie-Tooth disease, axonal, type 2O	614228	*DYNC1H1*	AD	Delayed motor milestones; distal lower limb weakness; paresthesias; neuropathic pain	[66,67]
Perry syndrome	168605	*DCTN1*	AD	Parkinson’s disease; Depression; Weight loss; Hypoventilation; Frontotemporal dementia; Progressive supranuclear palsy	[68,69,70,71,72]
Pick disease of brain	172700	*MAPT; PSEN1*	AD	Behavioral disinhibition; Social behavior deficits; Progressive non-fluent aphasia	[73,74,75,76,77,78,79]
Supranuclear palsy, progressive, 1	601104	*MAPT*	AD	Parkinsonism; Supranuclear gaze palsy; Postural instability; Axial rigidity; Dysarthria	[74,75,80,81,82,83]

AD: autosomal dominant; *DCTN1*: dynactin 1; *DCX*: doublecortin; *DYNC1H1*: dynein, cytoplasmic 1, heavy chain 1; *KIF1A*: kinesin family member 1A; *MAPT*: microtubule-associated tau protein; *PAFAH1B1*: platelet-activating factor acetylhydrolase, isoform 1B, alpha subunit; *PSEN1*: presenilin-1; *TUBA1A*: tubulin, alpha-1A; *TUBB2A*: tubulin, beta-2A; *TUBB3*: tubulin beta-3; *TUBB4A*: tubulin, beta-4A; *TUBB4B*: tubulin, beta-4B; XL: X-linked.

**Table 2:** Primary cytoskeletal disorders involving actin and actin-related proteins with neurological manifestations.

Disease	OMIM	Gene(s)	Inheritance	Clinical Features	References
Baraitser-Winter syndrome 1	243310	*ACTB*	AD	Craniofacial and skeletal dysmorphic features; Ptosis and/or coloboma; Developmental delay or intellectual disability; Epilepsy; Structural brain abnormalities	[20,91]
Baraitser-Winter syndrome 2	614583	*ACTG1*	AD	Craniofacial dysmorphism; Ocular anomalies; Developmental delay; Epilepsy; Cortical malformations	[90,91]
Amyotrophic lateral sclerosis 18	614808	*PFN1*	AD	Progressive upper and lower motor neuron degeneration; Weakness; Respiratory decline	[96,97]
Charcot-Marie-Tooth disease, dominant intermediate E	614455	*INF2*	AD	Gait disturbance; Muscle atrophy; Diminished deep tendon reflexes; Moderate sensorineural hearing loss; Progressive renal failure	[98,99]
Periventricular nodular heterotopia 1	300049	*FLNA*	XLD	Epilepsy; Periventricular nodular heterotopia; Variable cognitive involvement; Vascular or connective-tissue manifestations	[100,101]
Spinocerebellar ataxia 5	600224	*SPTBN2*	AD	Slowly progressive cerebellar ataxia with gait ataxia; Limb incoordination; Dysarthria; Ocular motor abnormalities	[102,103]
Developmental and epileptic encephalopathy 5	613477	*SPTAN1*	AD	Early-onset epileptic encephalopathy; Infantile spasms or refractory seizures; Severe developmental delay; Microcephaly; Hypomyelination; Cerebral/cerebellar or brainstem atrophy; Spasticity	[104,105]

AD: autosomal dominant; *FLNA*: filamin, A; *PFN1*: profilin 1; *SPTAN1*: nonerythrocytic alpha-spectrin 1; *SPTBN2*: nonerythrocytic beta 2 spectrin; XLD: X-linked dominant.

**Table 3:** Primary cytoskeletal disorders involving intermediate filaments, neurofilaments, and cytoskeletal linker proteins with neurological manifestations.

Disease	OMIM	Gene(s)	Inheritance	Clinical Features	References
Epidermolysis bullosa simplex 5B, with muscular dystrophy	226670	*PLEC1*	AR	Muscle weakness; Brain atrophy; Delayed motor milestones; Psychiatric manifestations; Hemorrhagic blisters	[31,106,107,108,112]
Alexander disease	203450	*GFAP*	AD	Megalencephaly; Spasticity; Dementia	[21,115,116]
Hereditary sensory autonomic neuropathy type VI	614653	*DST*	AR	Distal sensory loss; significant pain insensitivity; Autonomic dysfunction	[117,118]
Charcot-Marie-Tooth disease, demyelinating, type 1F	607734	*NEFL*	AD; AR	Muscle atrophy and weakness; Sensory loss; Pes cavus; Diminished deep tendon reflexes	[119,120]
Charcot-Marie-Tooth disease, axonal, type 2E	607684	*NEFL*	AD	Muscle atrophy and weakness; Gait disturbance; Pes cavus	[121,122]
Giant axonal neuropathy 1, autosomal recessive	256850	*GAN*	AR	Proximal and distal muscle weakness; Cerebellar ataxia; Sensory impairment; Dysarthria; Dysphagia; Optic atrophy; Intellectual disability; Respiratory failure; Kinky hair; Ichthyosis; Keratosis pilaris	[22,125]

AD: autosomal dominant; AR: autosomal recessive; *DST*: dystonin; *GAN:* gigaxonin; *GFAP*: glial fibrillary acidic; *NEFL*: neurofilament light chain; *PLEC1*: plectin 1.

## Data Availability

No new data were created or analyzed in this study. Data sharing is not applicable to this article.

## Abbreviations

The following abbreviations are used in this manuscript:

3R-tau	three-repeat tau
4R-tau	four-repeat tau
ACTB	actin-beta
ACTG1	actin-gamma 1
AD	autosomal dominant
ALS18	amyotrophic lateral sclerosis 18
AMPA	alpha-amino-3-hydroxy-5-methyl-4-isoxazolepropionic acid
AR	autosomal recessive
Arp2/3	actin-related protein 2/3
ATP	adenosine triphosphate
BRWS1	Baraitser–Winter syndrome 1
BRWS2	Baraitser–Winter syndrome 2
CDCBM5	cortical dysplasia, complex, with other brain malformations 5
CFEOM1	congenital fibrosis of the extraocular muscles 1
CFEOM3A	fibrosis of extraocular muscles, congenital, 3a, with or without extraocular involvement
CMT	Charcot–Marie–Tooth disease
CMT1	Charcot–Marie–Tooth disease type 1
CMT1F	Charcot–Marie–Tooth disease, demyelinating, type 1F
CMT2	Charcot–Marie–Tooth disease type 2
CMT2E	Charcot–Marie–Tooth disease, axonal, type 2E
CMT2O	Charcot–Marie–Tooth disease, axonal, type 2O
CMTDIE	Charcot–Marie–Tooth disease, dominant intermediate E
CNS	central nervous system
DCTN1	dynactin subunit 1
DCX	doublecortin
DEE5	developmental and epileptic encephalopathy 5
DNA	deoxyribonucleic acid
DST	dystonin
DYNC1H1	dynein cytoplasmic 1 heavy chain 1
EBS5B	epidermolysis bullosa simplex 5B with muscular dystrophy
FEOM3	fibrosis of the extraocular muscles 3 locus
FLNA	filamin A
GAN	gigaxonin
GAN1	giant axonal neuropathy 1, autosomal recessive
GFAP	glial fibrillary acidic protein
GTP	guanosine triphosphate
HLD6	leukodystrophy, hypomyelinating, 6
HSAN VI	hereditary sensory and autonomic neuropathy type VI
INF2	inverted formin 2
kDa	kilodalton
KIF1A	kinesin family member 1A
KIF21A	kinesin family member 21A
LCAEOD	Leber congenital amaurosis with early-onset deafness
LIS1	lissencephaly 1
LIS3	lissencephaly 3
LISX1	lissencephaly, X-linked, 1
MAP	microtubule-associated protein
MAPT	microtubule-associated protein tau
MBP	myelin basic protein
MRI	magnetic resonance imaging
NCV	nerve conduction velocity
NEFL	neurofilament light chain
NESCAV	neurodegeneration and spasticity with or without cerebellar atrophy or cortical visual impairment
OMIM	online Mendelian inheritance in man
PAFAH1B1	platelet-activating factor acetylhydrolase 1B regulatory subunit 1
PFN1	profilin 1
PLEC1	plectin 1
PLP1	proteolipid protein 1
PNS	peripheral nervous system
PSNP1	supranuclear palsy, progressive, 1
PVNH1	periventricular nodular heterotopia 1
RNA	ribonucleic acid
SBH	subcortical band heterotopia
SCA5	spinocerebellar ataxia 5
SPTAN1	spectrin alpha, non-erythrocytic 1
SPTBN2	spectrin beta, non-erythrocytic 2
TDP-43	transactive response DNA-binding protein 43 kDa
TUBA1A	tubulin alpha 1A
TUBB2A	tubulin beta 2A
TUBB3	tubulin beta 3
TUBB4A	tubulin beta 4A
TUBB4B	tubulin beta 4B
XL	X-linked
XLD	X-linked dominant
